# Is the Erich arch bar the best intermaxillary fixation method in maxillofacial fractures? A systematic review

**DOI:** 10.4317/medoral.20448

**Published:** 2015-06-02

**Authors:** Saulo-Gabriel Falci, Dhelfeson-Willya Douglas-de-Oliveira, Paulo-Eduardo-Melo Stella, Cássio-Roberto Rocha-dos Santos

**Affiliations:** 1 PhD. DDS. PhD. Department of Oral and Maxillofacial Surgery, School of Dentistry, Federal University of Vales do Jequitinhonha e Mucuri, Diamantina, MG, Brazil; 2 MSc. PostGraduate Program in Dentistry, Federal University of Minas Gerais, Belo Horizonte, MG, Brazil

## Abstract

**Background:**

Intermaxillary fixation is used to achieve proper occlusion during and after oral and maxillofacial fracture surgery. The aim of this systematic review was to compare Erich arch bar fixation with other intermaxillary fixation methods in terms of the operating time, safety during installation, oral health maintenance and occlusal stability.

**Material and Methods:**

An electronic online search was conducted of the Scirus, PubMed, Ovid, Cochrane Library and VHL databases. A clinical trial dating from the inception of the data bases until August 2013 was selected. Studies that compared Erich arch bars with other intermaxillary fixation methods in patients older than 18 years-old were included. The studies were assessed by two independent reviewers. The methodological quality of each article was analyzed.

**Results:**

Nine hundred and twenty-five manuscripts were found. Seven relevant articles were analyzed in this review. The risk of bias was considered moderate for four studies and high for three clinical trials.

**Conclusions:**

There is not enough evidence to conclude that the Erich arch bar is the best intermaxillary fixation method in cases of oral and maxillofacial fractures.

**Key words:** Facial injuries, jaw fixation techniques, mandible, maxilla.

## Introduction

Open reduction-internal fixation (ORIF) is the precise anatomic reduction of a fracture, achieving proper occlusion and an early return to function (1). Before the development of ORIF, oral and maxillofacial fracture was treated basically by inter maxillary fixation for about 4 to 6 weeks. Thus, inter maxillary fixation methods were developed to improve this situation. Nowadays, with the conception of ORIF, the crucial goal of modern maxillofacial surgery is to achieve the highest possible quality of life by returning the patient to the best possible condition (2). This situation includes the shortest postoperative inter maxillary fixation possible. Thus, inter maxillary fixation is basically being used intraoperatively.

The Erich arch bar (EAB) and eyelets wire were the most commonly used methods of inter maxillary fixation prior to the conception of ORIF. In 1989, inter maxillary fixation screws (IFS) were developed to substitute the EAB (3). Studies assessing the performance of IFS reported that this fixation method could decrease the operating time and reduce the risk of needle stick-type injuries (4). Furthermore, IFS exhibited better gingival health maintenance than arch bars (5). In order to provide better inter maxillary fixation than EAB, other substitute methods have been described, including Leonard Buttons (1), Embrasure Wires (6), the Resin Bonded Arch Bar (7) and Dimac wires (8).

Oral and maxillofacial surgery research has been conducted to decrease the time of application and achieve better results. Studies assessing the performance of inter maxillary fixation of other methods in comparison to EAB have been performed (1,6,8). Therefore, the aim of this systematic review was to compare EAB fixation with other inter maxillary fixation methods in terms of the operating time, safety during installation, oral health maintenance and occlusal stability.

## Material and Methods

This study did not require ethics committee approval because it was a review without involvement of human participants or animals.

- Focus Question

Is the EAB the best oral and maxillofacial fixation method for patients with oral and maxillofacial fractures?

- Search Strategy

The research was carried out based on human research studies that compared the EAB with other inter maxillary fixation methods. Electronic searches were performed using the following databases: SCIRUS (MEDLINE/PubMed; science direct; PubMed, Central; Biomed); PubMed; OVID; Cochrane Library (systematic reviews; quality analyzes abstracts); CCRCT – (Cochrane Central Register of Controlled Trials); VHL (Virtual Health Library - LILACS, IBECS, MEDLINE and Scielo).

The keywords were searched in DeCs (Health Sciences Descriptors) and Mesh (Medical Subject Headings) and the following terms were used: (jaw fixation techniques* OR inter maxillary fixation* OR maxillomandibular fixation* OR maxillofacial fixation*) AND (erich bar* OR arch bar* OR erich arch bar). To identify studies of interest for this review, a general search strategy was adapted to the characteristics of each database. All papers and abstracts published in English, Spanish and Portuguese up to August 2013 were considered for assessment.

- Study Selection

For this systematic review, inclusion of the manuscripts was based on an analysis of the title and abstract of studies in relation to the eligibility criteria listed below.

- Type of study

Studies comparing EAB with other inter maxillary fixation methods. Selected papers were prospective and retrospective clinical trials.

- Participants 

Patients were ≥ 18 years old, submitted to treatment of oral and maxillofacial facture.

- Intervention

Studies comparing inter maxillary fixation using the EAB with other inter maxillary fixation methods.

- Exclusion criteria

Case reports, review articles, editorial or opinion articles, studies with no comparison of the fixation methods, and those with no available abstract on the databases were excluded from this systematic review.

- Review Method

The study selection process was performed by two reviewers independently (SGMF and DWDO) in two phases. First, the two reviewers identified all relevant studies through an electronic search by reading the titles based on the eligibility criteria. In the second phase, the preselected studies were analyzed by the same two reviewers. When necessary, the authors of the RCTs were contacted by e-mail to clarify issues related to the trials. Disagreements were solved by consensus between the two reviewers. Each researcher qualitatively assessed the studies using an assessment form. Data were collected for the following items: 1) author; 2) year of publication; 3) study design; 4) inter maxillary fixation methods; 5) origin and 6) results regarding: (a) the time required for application; (b) Needle-stick injuries; (c) periodontal damage or hygiene index and; (d) inter maxillary fixation stability.

A methodological quality assessment of the studies was performed based on the revised recommendations of the Consolidated Standards of Reporting Trial (CONSORT) statement (9) and a previous quality was estimated for each study based on a published study about systematic reviews (10) and a previous systematic review (11): low (< 4 points), moderate (4 - 6 points) and high methodological quality (> 6 points)  Table 1.

**Table 1:**
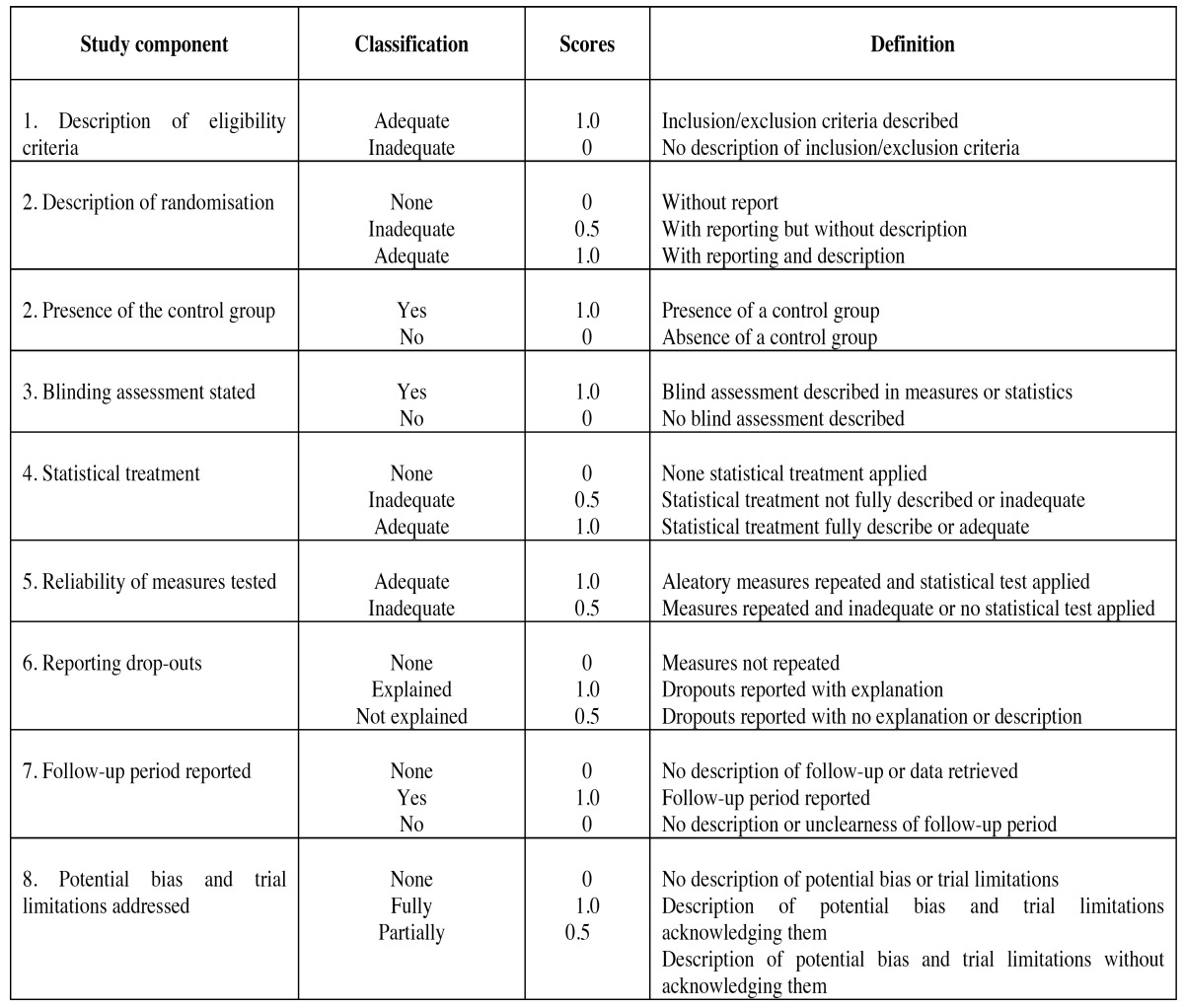
Criteria for assessing the quality of included studies.

## Results

**Figure 1:**
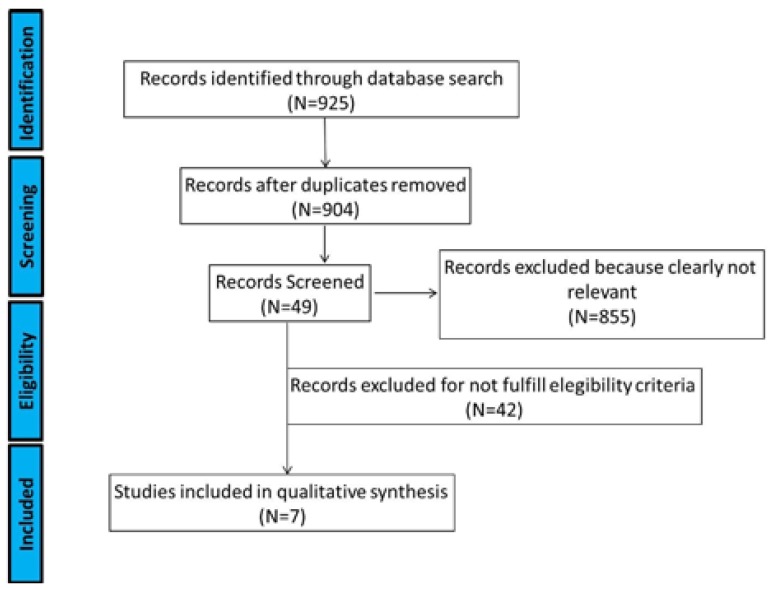
Flowchart for search results.

A total of 925 manuscripts were found in the described databases. After eliminating duplications, the electronic search yielded 904 references. A total of 855 references were removed based on the assessment of the title and abstract. The full texts of the remaining 49 articles were read. Finally, 42 articles were removed, after reading the full text, as they did not fulfill the eligibility criteria. Thus, a total of 7 studies met the selection criteria and qualified for the final analysis (Fig. 1). Three studies were conducted in India (4,7,12), two in the United Kingdom (1,8), one in the United States (6) and one in South Korea (13). All studies compared the EAB with other fixation methods. The other fixation methods were: IFS in three studies (4,12,13), the Leonard Buttons (1), the Resin Bonded Arch Bar (7), Embrasure Wires (6) and Dimac Wires (8). Five studies were prospective (4,7,8,12,13) and two were retrospective (1,6).

The time required for the application of EAB was greater than for IFS (*p*<0.001) (4,13), Leonard Buttons (*p*=0.013) (1) and Dimac Wires (*p*<0.05) (8). Needle-stick injuries were less common with Dimac wire fixation than with EAB fixation (*p*<0.05) (8). The other articles did not assess needle-stick injuries (1,4,6,7,12,13). The results showed the greatest gingival and plaque index and the worst oral hygiene in the EAB group (*p*<0.05) (13), (*p*<0.001) (4), (*p*=0.05) (1). Inter maxillary fixation stability and the occlusion index was better in Leonard Buttons fixation than in EAB fixation (*p*=0.027) (1). On the other hand, IFS showed no difference regarding inter maxillary stability and the occlusion index immediately after inter maxillary device installation (4,13). Two and four weeks after installation, the EAB exhibited better occlusal stability than IFS (4,12,13). Four articles were considered to have a moderate risk of bias (1,4,8,13) and three were classified as high risk for bias ( Table 2) (6,7,12).

**Table 2:**
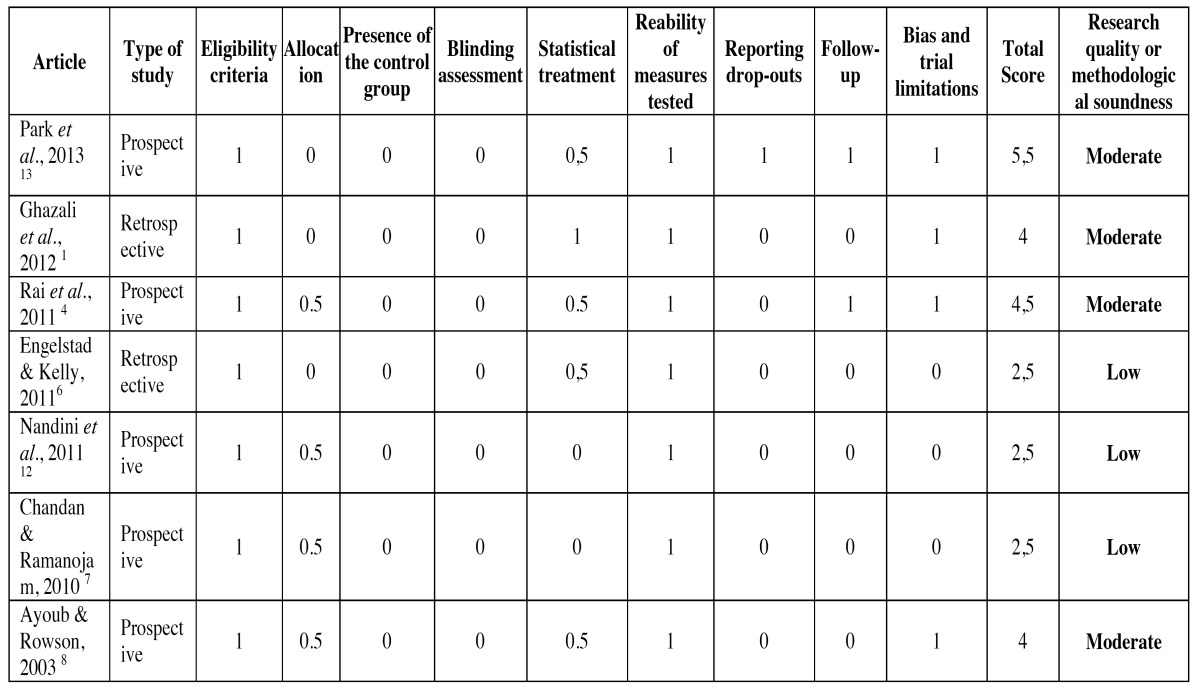
Quality assessment of the studies included.

Of the articles that had a moderate risk of bias, two compared the EAB with IFS (4,13), one compared the EAB with Leonard Buttons (1) and one compared the EAB with Dimac Wires (8).

## Discussion

Before the development of plates and screws for the fixation of facial fractures, most maxillofacial fractures were treated almost exclusively by closed reduction with inter maxillary fixation. The EAB was regarded as the gold standard method of inter maxillary fixation because it promoted better occlusal stability than the other methods available. The inter maxillary fixation time was about four to six weeks, and the inter maxillary fixation method should be stable during all this time. Nowadays, the management of mandibular fractures is the Open Reduction & Internal Fixation (ORIF). This technique allows shorter inter maxillary fixation period minimizing patients’ inconvenience. In this situation inter maxillary fixation has been used almost exclusively during the trans-operative period, allowing the patient to keep the mouth opened after surgery and to recover routine in a short period of time (2). This technique, where possible, should be selected by the surgeon. Thus, inter maxillary fixation stability is required almost exclusively during surgery. On the other hand, in some clinical situations, such as unstable fractures or in doubt of the quality of ORIF, the inter maxillary fixation must be extended after surgical procedure. In these cases, the results of this systematic review suggest that the EAB exhibits better results when prolonged inter maxillary fixation is required.

Recent studies have reported some disadvantages of EAB application such as the long operating time, needle-stick injuries, the high plaque index, periodontal damage, movement of the teeth in lateral and extrusive direction (1,4,8,12,13). Furthermore, in some clinical situations such as anterior open bite is present, in pediatric fractures, patients with mental disorders, and in partial and completely edentulous fractures, EAB should be avoided (1). Thus, alternative methods, like the IFS, have been developed to eliminate these disadvantages and promote occlusal stability during the operating time. Since 1989, this inter maxillary fixation method has been used for this purpose (3). This fixation method eliminated needle-stick injuries and decreased the operating time, as well as favoring better gingival health maintenance (4,5). However, this method also has limitations, such as iatrogenic root injuries, screw fractures, mucosal coverage of the screw and screw loosening (14-17). This systematic review selected three papers which compared IFS with the EAB. One of these papers was of low quality with a high risk of bias (12). The others were of moderated quality (4,13). Both of them indicated that the EAB involved a longer operating time and worse hygiene maintenance than IFS. On the other hand, EAB was better than IFS in terms of postoperative occlusal stability. The IFS were lost in 16.67% of cases and the application of this technique caused root damage in 5.83% of cases (4).

The use of Leonard Buttons is another inter maxillary fixation method that has been compared with the EAB (1). Although the authors state that this research is a pilot study, they found equally good reduction when comparing it with the EAB. Furthermore, Leonard Buttons can be installed faster than the EAB and can provide better oral hygiene maintenance. Leonard Buttons are attached to the tooth by steel wires, like the EAB. In this study, needle-stick injuries were not reported during the installation of Leonard Buttons (1).

With regards to the other fixation methods reported in this systematic review, Dimac Wires exhibited a shorter operating time than the EAB (*p*<0.05) (8). Needle-stick injuries were reduced because the end of the wires was secured with an artery forceps whilst passing the wire between teeth. Like Leonard Buttons, Dimac Wires are only installed in the premolar and molar regions, which improves oral hygiene maintenance when compared with the EAB (8).

The results of this systematic review found that the relevant studies exhibited a moderate and high risk of bias. No study with a low risk of bias has been found. None of the selected studies involved a control group or a blinding assessment. Only one study reported dropouts and only two studies reported the follow-up. These findings lead to the conclusion that the methodological quality of studies about inter maxillary fixations methods needs to improve. Nowadays, evidence-based practice is mandatory in clinical decision-making and the research must be done with a low risk of bias following guidelines, such as the CONSORT (9) statement, as was performed in this systematic review.

A protocol was employed to guide the search strategy, study selection and data collection. However, the present systematic review may have potential limitations. Firstly, a selection bias may have occurred, since the search was restricted to publications in the Portuguese, Spanish and English languages. Secondly, no hand search of published studies was performed. Finally, meta-analysis was not possible. Well-conducted randomized controlled trails and long-term postoperative follow-ups are required to corroborate or refute the findings of this systematic review.

In the assessed articles, EAB application was associated with increases in the operating time, the plaque index and the chances of a needle-stick injury when compared with other inter maxillary fixation methods. Regarding occlusal stability, the inter maxillary fixation methods are similar during surgery and in the postoperative period. However, when a prolonged inter maxillary fixation is required, the EAB provides better occlusal results than other inter maxillary fixation methods.

## Conclusions

Due the quality of studies assessed the authors concluded that there is not enough evidence to suggest that the EAB is the best or worst inter maxillary fixation method in cases of oral and maxillofacial fractures. The results and conclusions of the present systematic review must be viewed with caution, as none of the studies reviewed had a low risk of bias. Thus, the reliability of the results could be questionable.
